# Resolutions—Dental Education

**Published:** 1888-08

**Authors:** 


THE following resolution looking toward instructing the National Association of Dental Faculties was unanimously passed by the union meeting of the Conn. Valley and Mass. Dental Societies :

WHEREAS, The standard of dental education is constantly advancing, and the demands for higher attainments made upon the members of the profession are becoming greater each year ; and,

WHEREAS, The time required to complete the course of instruction in most of our colleges is so short that it is impossible for the average student to meet the requirements and do justice to himself ; and,

WHEREAS, It has been proven feasible by actual experiment in several of our dental colleges to lengthen the time requisite for graduation ; therefore, in the interests of higher education and uniformity in the degree, be it

*Resolved*, That it is the sense of this union meeting of the Connecticut Valley and Massachusetts Dental Societies that the National Association of Dental Faculties be, and is hereby requested, to adopt as a requirement for graduation, three full years' study, two at least of which shall be spent attending lectures, in separate years, at some regular school, whose required term shall not be less than seven months.

A similar resolution was also passed by the New Jersey State Society, and the Maine Dental Society. The Illinois State Society has also passed a resolution recommending a three years' course. These general expressions of the leading societies in the country from the far east to the Mississippi Valley should have an influence upon the action of the National Association of Dental Faculties.

---

AT the union meeting of the Connecticut Valley and Massachusetts Dental Societies the following resolutions were introduced, and unanimously adopted :

WHEREAS, Certain manufacturers and dealers in dental instruments and materials have formed a combination known to the profession as the Dental Trade Association ; and,

WHEREAS, The forming of any such combination can only be an obstacle, retarding progress in the direction of higher professional attainment,

*Resolved*, That we consider the forming of such combinations to be a reflection on the scientific and professional character of our profession.

*Resolved*, That we invite all members of said combination to withdraw from the combination, and we pledge them our hearty interest and support.

